# Assessing the effect of *Alpinia galanga* extract on the treatment of SSRI-induced erectile dysfunction: A randomized triple-blind clinical trial

**DOI:** 10.3389/fpsyt.2023.1105828

**Published:** 2023-04-18

**Authors:** Farzad Akbarzadeh, Mahboubeh Eslamzadeh, Ghazal Behravan, Alireza Ebrahimi, Seyed Ahmad Emami, Atefe Gilan, Najme Sadat Hoseinian

**Affiliations:** ^1^Department of Psychiatry, Faculty of Medicine, Mashhad University of Medical Sciences, Mashhad, Iran; ^2^Department of Pharmacology, Faculty of Medicine, Mashhad University of Medical Sciences, Mashhad, Iran

**Keywords:** SSRI (selective serotonergic reuptake inhibitors), sexual dysfunction, erectile dysfunction, *Alpinia galanga*, libido, side effect, clinical trial

## Abstract

**Objective:**

SSRIs are considered the first line in the medical treatment of depression and anxiety disorders. One of their most common side effects, sexual dysfunction, has led many patients to discontinuing their medication and treatment course. *Alpinia galanga*, a plant from the ginger family, has been shown to enhance androgenic activity and sexual function. This study aimed to assess whether the addition of *Alpinia galanga* extract to the treatment regimen of adult males consuming SSRIs can improve SSRI-induced erectile dysfunction.

**Materials and methods:**

This triple-blind randomized clinical trial was conducted on 60 adult males who were being treated with SSRIs at the time of the study. The participants were divided into two groups, a group of 30 people receiving 500 mg of *Alpinia galanga* extract and a group of 30 subjects receiving placebo. The population were re-assessed on week 2 and week 4 of the study using the international index of erectile function (IIEF), the Beck Depression Inventory, and the Beck Anxiety Inventory. In all the tests, a *p*-value of 0.05 was considered as the cut-off for significance.

**Results:**

At the beginning of the study, the IIEF scores of the placebo group and the intervention group were 10.6 ± 3.8 and 11.2 ± 4.8, respectively, which were not significantly different (*p*-value = 0.577). By week 4 of the study, the IIEF scores of the control group and the *Alpinia galanga* group had increased to 13.7 ± 4.3 and 17.4 ± 3.7 respectively, which demonstrates a remarkably larger increase in the group receiving *Alpinia galanga* extract in comparison to the placebo group (*p*-value < 0.001).

**Conclusion:**

In this study, the effect of the addition of *Alpinia galanga* extract to the treatment regimen of male patients using SSRIs on the sexual dysfunction experienced by this group has been promising. Similar results, if proven, can aid both patients and clinicians in making and following better treatment plans with more pleasant outcomes.

**Clinical trial registration:**

[https://clinicaltrials.gov/], identifier [IRCT20101130005280N41].

## Introduction

Selective serotonin reuptake inhibitors (SSRIs) are the most commonly used antidepressants and one of the most frequently prescribed medications worldwide (1). The higher tolerability and overall safety of SSRIs in comparison with older antidepressants, have made them a common and popular component of the treatment regimen of disorders such as depression and anxiety (2). SSRIs are used to treat a variety of psychiatric conditions and mental disorders including major depressive disorder, obsessive–compulsive disorder (OCD), anxiety disorders including generalized anxiety disorder (GAD), panic disorder, social anxiety disorder, post-traumatic stress disorder (PTSD), eating disorders, chronic pain, phobias, and depersonalization disorder. In addition, they are also used off-label in the treatment of other disorders such as irritable bowel syndrome, fibromyalgia, premenstrual syndrome, and chronic pain disorder (3). SSRIs have been shown to significantly reduce mortality and morbidity rates associated with depression (4). The diverse therapeutic effect of SSRIs can be explained taking into consideration their mechanism of action, which is mainly increasing serotonin levels in certain synapses and brain regions as a result of somatodendritic 5HT1A autoreceptor desensitization. It is hypothesized that their side effects are due to an increase in serotonin levels in specific serotonin receptor subtypes in other regions of the body where the regulation of physiological processes occur (5). SSRIs have also been associated with side effects including nausea and vomiting, headaches, diarrhea and/or constipation, drowsiness, blurred vision, insomnia, agitation, dizziness, serotonin syndrome, bruxism, bipolar switch, akathisia, weight loss and/or weight gain, as well as sexual side effects such as a decrease in libido, erectile dysfunction, dysorgasmia, and anorgasmia (6). Regardless of how bothersome certain experienced side effects may be, only around 40% of patients report these side effects to their prescribing physician; this is even more significant for sexual side effects (7). The prevalence of sexual dysfunction among SSRI consumers has been reported to be as high as 30 to 50% (8). SSRI-induced sexual dysfunction is often temporary, and resolves after the discontinuation of the medication. A small percentage of patients, however, experience post SSRI dysfunction, a condition in which sexual dysfunction persists even after treatment termination (9).

Sexual behavior and desire is regulated by both cortical areas of the human brain as well as subcortical structures including the hypothalamus, spinal cord, and brainstem. At the central level, serotonergic as well as dopaminergic systems have been shown to play a significant role. In addition, cholinergic, adrenergic, and other transmitter systems contribute to this function. Loss or decrease in proper sexual function has been shown to significantly impair the quality of life of affected individuals as sexual well-being is considered one of the most important aspects of a person’s quality of life (10). Patients being treated with SSRIs should routinely be asked about their sexual function in order to be able to identify such problems early. Medication-induced sexual dysfunction, if ignored, may lead to non-compliance, decrease in patient compliance, relapse, compromisation of treatment outcomes, lower quality of life, and lengthening of depressive episodes, which significantly contradict treatment goals in depressed patients (11). A variety of pharmacological and non-pharmacological methods have been proposed to manage SSRI-induced symptoms of sexual dysfunction. Methods such as the “wait and observe” approach and “drug holiday” have been used in the management of SSRI-associated sexual dysfunction. Some studies have reported withdrawal syndrome and a disturbance in therapeutic efficiency caused by these management methods (12). It has also been reported that switching the SSRI medication can result in a decrease in sexual side effects (13). In the treatment of psychiatric disorders, the “augmentation approach” has also been used to enhance medication efficacy as well as decrease certain side effects. Some studies have reported a significant increase in patients’ libidos as a result of bupropion augmentation treatment. In addition, exercise has been shown to improve sexual function and desire due to the activation of sympathetic nervous system (14). Despite the use and practice of the various methods mentioned, most patients (80%) report little or no improvement after 6 months of treatment. Given the serious and harmful effects of depression and untreated mental disorders on the affected individual, the necessity to treat mental illnesses properly and adequately, as well as the wide use of SSRIs for this means, it is necessary and crucial to investigate and use efficient methods to reduce the inevitable side effects of these medications to the highest possible extent (15). Loss of sexual function and libido has been reported to affect 25–50% of patients with unipolar depression (16). Therefore, the proper management of sexual function in depressed patients is of high importance. *Alpinia galanga*, also called blue ginger, is a plant in the ginger family which has been used as a herb in Unani medicine and as a spice in many traditional cookeries, including Southeast Asian cuisine. It has widely been used to treat microbial infections, rheumatic pains, fever, dyspnea, gastritis, inflammations, and otitis internal. The major compound isolated from *Alpinia galanga* with various biological functions is 1′S-1′-acetoxychavicol acetate (ACE). Anti-fungal, anti-inflammatory, anti-malarial, and anti-oxidant activity, as well as anti-diabetic and anti-ulcer properties have been attributed to *Alpinia galanga* (17).

Another study conducted by Ratnasooriya et al. concluded that rhizomes of *Alpinia calcarata* Roscoe significantly increased the erectile ability as well as the serum testosterone levels in male rats. However, a slight impair in sexual motivation in the partner preference test was observed (18). A study on 40 rats conducted by Negm et al. also reported that *Alpinia officinarum* (known as lesser galanga, another plant from the ginger family) significantly increased testosterone serum levels, follicular stimulating hormone (FSH), luteinizing hormone (LH), and superoxide dismutase (SOD) levels. They inferred that *Alpinia officinarum* could potentially lead to an improvement in human fertility as well as sexual function (19).

Some studies have reported increases in androgenic activity as well as improvements in sexual function and libido as a result of *Alpinia galanga* (18). In this study, we aimed to assess the possible effects of *Alpinia galanga* extract on the erectile function and medication-induced sexual side effects of adult male patients under treatment with SSRIs.

## Materials and methods

This randomized triple-blind clinical trial study was conducted on 60 patients diagnosed with anxiety and/or depression who were being treated with SSRI medications. This project was approved by the “Research Ethics Committee of Mashhad University of Medical Sciences” on 25 May 2021 under the code IR.MUMS.MEDICAL.REC.1400.189. The participants were all male consumers of SSRI medications who complained of new-onset sexual dysfunction; they were selected through convenience sampling method and were referred to outpatient psychiatric clinics of Ibn-Sina, Imam Reza, and Ghaem Hospitals in Mashhad, Iran from March 2020 to March 2021.

Considering the fact that no clinical study has previously investigated the possible effect(s) of the *Alpinia galanga* extract on SSRI-associated male sexual dysfunction, this project was considered a pilot study, and therefore, a minimum of 30 participants were assigned to each of the two groups. Thus, a total number of 60 people were selected as the sample population. Informed oral and written consent was obtained from all participants after the project was thoroughly explained to them and the participants were assured they were free to leave the study at any time that they wished.

In this triple-blind clinical trial, the participants, the data collectors, as well as the data analysts were blinded. The participants were chosen through convenience sampling method and randomly divided into two groups using a computerized randomizer. The permuted block randomization technique was used to divide the participants into two equal groups, each consisting of 30 subjects.

### Inclusion criteria

The inclusion criteria for the study were as follows: male gender, age under 60 and over 18, use of SSRI medication for at least the past 6 consecutive weeks, sexual dysfunction (confirmed by examiner physician’s diagnosis), lack of substance abuse, lack of co-existing other mental illnesses (as ruled out by a clinical psychiatrist), lack of physical/somatic illnesses, and lack of using medications other than SSRIs.

The exclusion criteria for the study included: symptoms of liver, kidney, or thyroid dysfunction, use of any medication other than SSRIs, age 18–60, substance use disorder, other somatic illnesses, co-existing mental disorders other than major depression or anxiety. In addition, the participants were routinely checked during the study and those who were found to meet any of the above criteria were removed from the sample population.

### Medications

One group received *Alpinia galanga* extract in the form of a 500-mg tablet, while the other received similar looking placebo tablets containing Avicel (a starch-like substance). Both groups were told to consume their medication with a glass of milk on an empty stomach. The participants were assessed on week 2 and week 4 of the study. Additionally, in order to investigate the possible side effects, the participants were regularly assess using a self-report medication side effect questionnaire, as well as laboratory tests including complete blood count (CBC), blood urea nitrogen (BUN), Creatinine, alanine transaminase (ALT), aspartate transaminase (AST), alkaline phosphatase (ALP), and thyroid stimulating hormone (TSH).

### Questionnaires and evaluations

The International Index of Erectile Function (IIEF) was used to evaluate the participants. It is a widely used, multidimensional, self-report questionnaire used to assess the sexual function of males. The validity and reliability of this questionnaire has also been proven for the Iranian population (20). It contains 15 questions which assess different aspects of the test-taker’s sexual function such as erectile function, orgasmic function, sexual desire, intercourse satisfaction, and overall satisfaction over the past 4 weeks. There are 6 possible answers for every question and a score of 0–5 is awarded to each question. The total score is calculated by summing the score of each individual question. A maximum score of 75 can be obtained from this questionnaire with higher scores indicating better sexual function. A total score of 5–7, 8–11, 12–16, 17–21, and 22–30 indicate no dysfunction, mild dysfunction, mild to moderate dysfunction, moderate dysfunction, and severe dysfunction, respectively (21). The validity and reliability of this questionnaire has been approved for the Iranian population (22).

Throughout the years, different inventories and questionnaires have been used to measure the severity and prevalence of depression among populations. The Beck Depression Inventory (BDI) is one of the most widely used inventories for this means. It is a 21-question multi-choice self-report inventory. There are three versions of this inventory: BDI, BDI-1A, and BDI-II. The current version (BDI-II) was used in this study and is designed for ages 13 and up. This version was published in 1996 and takes into account physical as well as cognitive symptoms of depression. Previous studies have confirmed a high degree of reliability and validity for this inventory in the Iranian population (23). A value of 0 to 3 has been assigned for every answer to each question. The total score is calculated by summing up the scores attained in every question and can range from 0 to 63. Higher scores indicated higher levels of depression. The standard cut-off scores are as follows: A total score of 0–13, 14–19, 20–28, and 29–63 demonstrate normal, mild, medium, and sever levels of depression, respectively, (24). Its reliability and validity has been proven for the Iranian population (25).

The Beck Anxiety Inventory (BAI), a multiple-choice self-report inventory consisting of 21 questions, has been widely used to measure the prevalence and severity of anxiety in different studies. It has been designed for ages 17 and up and takes into consideration physical, cognitive, and emotional symptoms of anxiety in the last week such as numbness, palpitations, fear of future events, and fear of losing control of oneself. The reliability and persistence of the Beck Anxiety Inventory has been proven in Persian studies (26). In this 21-question inventory, each answer is scored on a scale value of 0–3. The total score can range from 0 to 63, with lower scores indicating lower anxiety levels. The standardized cut-offs used for interpretation are as follows: A total score of 0–7, 8–15, 16–25, and 26–63 indicate normal, mild, medium, and severe anxiety levels, respectively, (27). This inventory has been proven to be valid and reliable for use the Iranian population (28).

Data were analyzed using IBM SPSS 26.0 software using tests such as paired *t*-test and Chi-square. In all of the tests, 0.05 was considered as the cut-off for significance.

This clinical trial was registered under the code IRCT20101130005280N41 in the Iranian Registry of Clinical Trials.

## Results

This triple-blind randomized clinical trial study was conducted on 60 adult males who were under treatment with SSRI medications and had complained new-onset sexual side effects. The participants were randomly divided into two groups, one group receiving *Alpinia galanga*, while their counterparts received placebo.

Table 1 demonstrates the information of the two groups.

**Table 1:** Comparison of the placebo group and the *Alpinia galanga* group.

	Placebo group		*Alpinia galanga* group		*p*-value
Age [median and (range)]	43 (25–62)		45 (22–63)		0.865
Beck Depression Score [mean ± standard deviation]	Baseline	20.5 ± 6.7	Baseline	21.3 ± 5.9	0.657
	Week 2	17.3 ± 6.2	Week 2	17.4 ± 5.4	0.974
	Week 4	12.9 ± 7.5	Week 4	13.0 ± 4.6	0.962
Beck Anxiety Score [mean ± standard deviation]	Baseline	22.7 ± 8.9	Baseline	23.4 ± 4.7	0.851
	Week 2	18.1 ± 7.2	Week 2	17.7 ± 3.8	0.903
	Week 4	10.6 ± 6.2	Week 4	8.6 ± 4.2	0.456
IIEF Score [mean ± standard deviation]	Baseline	10.6 ± 3.8	Baseline	11.2 ± 4.8	0.577
	Week 2	11.4 ± 3.8	Week 2	14.1 ± 4.4	0.015
	Week 4	13.7 ± 4.3	Week 4	17.4 ± 3.7	0.001

There was no significant difference between the two groups in terms of age (*p*-value = 0.865). As shown in Table 1, there was no statistically significant difference between the Beck depression scores of the two groups at the beginning of the study, as well as by week 2, and week 4 of the study (*p*-value>0.05).

By the end of the study both groups had experienced a significant decrease in their Beck depression scores compared to the beginning of the project (*p*-value<0.001). In addition, the Beck depression scores of both groups were significantly lower by the end of the study compared to week 2 of the study (*p*-value<0.001). However, these changes were not significantly different among the two groups (*p*-value = 0.679).

No statistically significant difference was observed between group A and group B in terms of Beck anxiety score at the beginning of the study, as well as by week 2 and week 4 of the study (*p*-value>0.05). The Beck anxiety score had notably decreased in both groups by week 4 of the study compared to the beginning of the project (*p*-value<0.001). Moreover, both the control and the intervention group had obtained remarkably lower scores on the Beck anxiety inventory on week 4 of the study compared to the second week of the project (*p*-value<0.001). However, these changes were not significantly different among the two groups (*p*-value = 0.470).

**Figure 1:**
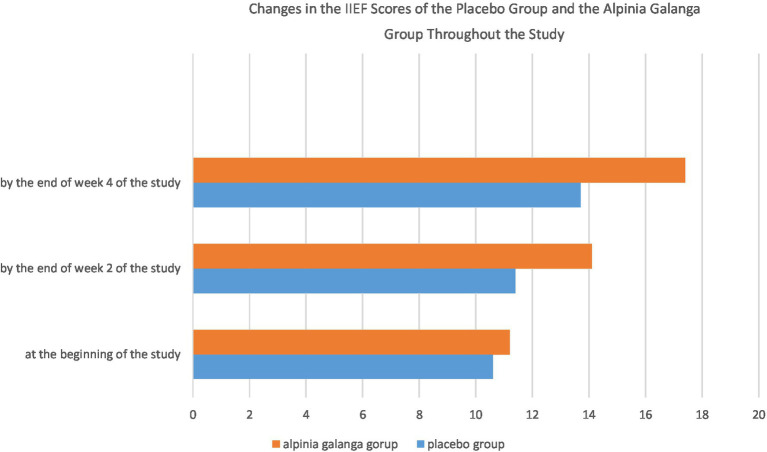
Changes in the IIEF scores of the placebo group and the *Alpinia galanga* group throughout the study.

The International Index of Erectile Function scores of the placebo group and the *Alpinia galanga* group at the beginning of the study were 10.6 ± 3.8 and 11.2 ± 4.8, respectively (mean ± standard deviation). The initial IIEF score was not significantly different between the two groups at the beginning of the study (*p*-value = 0.577). By week 2 and week 4 of the study, however, the *Alpinia galanga* group had obtained significantly higher marks on the IIEF compared to the placebo group (*p*-value<0.05; Figure 1).

The IIEF score of both groups had significantly increased by week 4 of the study in comparison to the beginning of the study (*p*-value<0.001). Furthermore, both groups had significant increases in their IIEF scores on week 4 of the study compared to week 2 of the project (*p*-value<0.001). In addition, the IIEF scores of both groups had significantly increased by the end of week 2 of the study compared to the beginning of the project (*p*-value<0.001).

Repeated measures design was applied to assess and compare changes in the IIEF scores of the two groups. Mauchly’s sphericity test (considering *w* = 0.673) and Greenhouse–Geisser correction (considering *ɛ* = 0.75) were used to evaluate the two groups. There was a notably remarkable difference among the two groups in terms of the change in their IIEF scores, with the *Alpinia galanga* group experiencing a significantly higher increase in their IIEF scores compared with their placebo-consuming counterparts (*p*-value<0.001).

No significant correlation was observed between changes in IIEF scores and changes in Beck Depression Inventory scores in both groups (*p*-value>0.05). In addition, there was no significant correlation between changes in IIEF scores and changes in Beck Anxiety Inventory scores of the two groups (*p*-value>0.05).

The two groups showed no significant changes in their AST, ALT, or ALP laboratory values (*p*-value>0.05). The creatinine level of the participants consuming *Alpinia galanga* at the beginning and end of the study was 0.78 ± 0.16 and 0.82 ± 0.21, respectively (*p*-value = 0.091). Considering the normal range of creatinine within 0.6–1.2 mg/dL (53–106 μmol/L) (29), the AST, ALT, ALP, and creatinine results of the participants were within the normal range throughout the study. There was no significant association between the laboratory values and the use of *Alpinia galanga* (Table 2)

**Table 2:** Laboratory values of the participants throughout the study.

	Placebo group	*p*-value	Intervention group	*p*-value
Primary AST	27.32 ± 11.82	0.236	24.65 ± 9.48	0.349
Week 4 AST	27.83 ± 12.13		23.96 ± 9.71	
Primary ALT	32.06 ± 10.27	0.455	27.17 ± 11.19	0.287
Week 4 ALT	32.67 ± 9.97		26.41 ± 11.52	
Primary ALP	96.74 ± 34.72	0.587	73.79 ± 32.57	0.385
Week 4 ALP	91.38 ± 34.39		72.55 ± 29.18	

**Figure 2:**
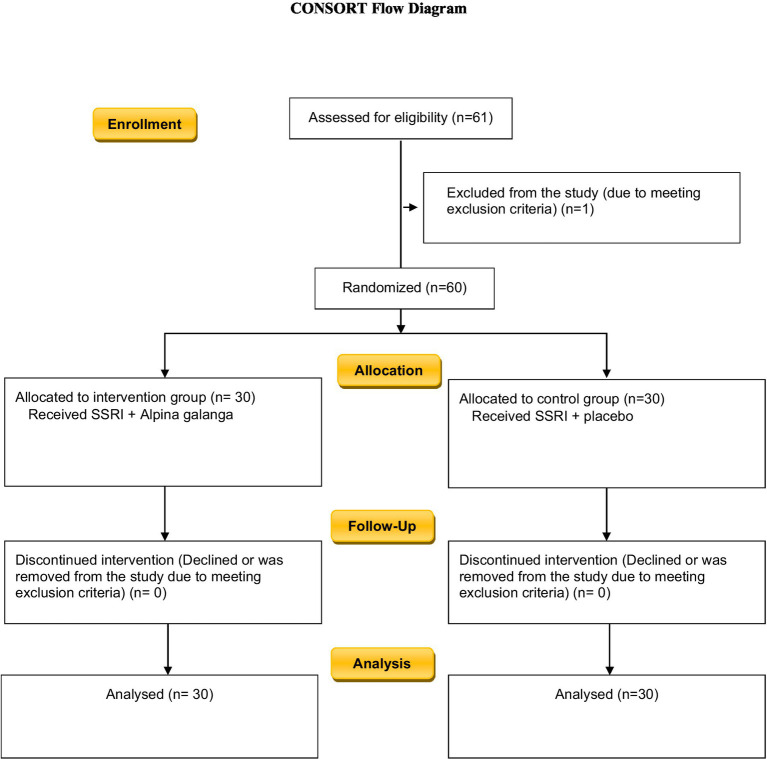
Study flow chart.

Effect sized was calculated using Cohen’s d statistic. Cohen’s *d* was equal to 0.49, which can indicate an acceptable effect size (Figure 2).

## Discussion

This triple-blind clinical trial aimed to assess the possible effects of *Alpinia galanga* extract on SSRI-associated male sexual dysfunction. Sixty male SSRI consumers were randomly assigned to two groups; one group consuming *Alpinia galanga* extract and the other receiving placebo. Both groups showed a significant decrease in their Beck Depression and Anxiety Inventory scores. This decrease was possibly due to the use of SSRI medications. The intervention group showed a notably higher increase in their IIEF scores by the end the study compared to the control group. Similar results, if proven by further studies, could suggest that *Alpinia galanga* may have a positive effect on enhancing male sexual dysfunction and may be used to reduce sexual side effects in patients consuming SSRI medications. Various other studies on both animals and human subjects have suggested sexual function enhancement as a result of *Alpinia galanga* use.

A 2021 narrative review study by Farahmand et al. also reported that plants of the ginger family may improve erectile function and augment sexual satisfaction. In addition, they concluded that plants such as Tribulus can increase testosterone levels as well as the number of sperms (30). This further suggests that plants of the Alpinia family may play a role in improving sexual function in males.

A 2019 literature review by Chen et al. found that 15% of heterosexual couples worldwide suffer from sexual disharmony, and that 40–50% of these cases are due to male sexual dysfunction factors. They wrote that the use of herbal medicine can be an effective method for reducing sexual dysfunction due to the increasing interest in the consumption of these medicines as well as their low cost and minimal side effects. They found that numerous herbal medications, including *Anacyclus pyrethrum*, *Anethum graveolens* L, and *Alpinia calcarata* Roscoe (from the ginger family) may serve as “aphrodisiac” agents as evident by their augmentation of erectile function and durability, sexual arousability, and sexual performance (31). These findings are in line with our results that herbal medications such as *Alpinia galanga* can play a role in improving male sexual function.

A review article by Banihani inferred that the use of ginger, or its derivatives, particularly in conditions of oxidative stress, enhances the production of testosterone. The presumed mechanism is that this effect is induced by the increase in luteinizing hormone (LH), reduction of lipid peroxidation and oxidative stress in the testes, increasing cholesterol levels, and enhancing blood flow to the testicles (32). These reports also align with our findings and provide a possible theory of the mechanism of action of plants of the ginger family in ameliorating erectile function.

A study by Qureshi et al. reported increases in sperm motility and sperm count as well as weight gain of the testes of mice being treated with *Alpinia galanga* and curcuma longa; the increases however were significantly higher in mice who had received *Alpinia galanga* extract (33). Similar to our study, their findings suggest that *Alpinia galanga* may play a role in the enhancement of sexual function.

A systematic review by da Cruz et al. found aphrodisiac effects for various herbal medications. They concluded that *Tribulus terrestris* and *Eurycoma longifólia* increase testosterone serum levels, ginseng stimulates smooth muscle relaxation with nitrous oxide, Lepidium meyenii improves sexual function, and that *Mondia whitei* (White’s ginger) improved libido and erection (34). The findings of this study are also in line with our results and suggest that plants of the ginger family (including *Alpinia galanga*) may play a role in the enhancement of sexual function, desire, and fertility.

A 2021 systematic review by Luft et al. on the pharmacological interventions in SSRI-associated sexual dysfunction reported that medications such as sildenafil and pycnogenol have been shown to enhance SSRI-induced sexual dysfunction in comparison with placebo (35). Some studies, including a systematic review by Maleki-Saghooni et al. have reported the positive effect of other herbal medications such as saffron (*Crocus sativus*) in the treatment of male sexual dysfunction (36). Further assessments are required on the use of herbal medications in treating erectile dysfunction as well as to compare their effectiveness and side effects.

This study was the first clinical trial to investigate the effects of the consumption of *Alpinia galanga* extract on the sexual side effects experienced by male patients being treated with SSRIs. As shown by a Cohen’s d of 0.49, the effect size was acceptable and a significant improvement was observed in the sexual function of the intervention group. Similar results, if proven, could lead to more effective treatment plans and higher compliance in psychiatric patients consuming SSRIs. In addition, due to the popularity of herbal medications and the participants’ enthusiasm, almost all the participants willingly completed the study and were content with their results and there was very little loss to follow-up.

It should be noted, however, that due to cultural and/or personal reasons, obtaining precise and accurate information about the sexual function of patients may be challenging. One of the limitations of this study could be that it was conducted in a limited group (heterosexual married males aged 18–60 who were being treated with SSRIs) and that the population was followed up for 4 weeks. Although significant changes were seen, we suggest future projects study similar possible effects in other groups such as females, the transgender population, people of other sexual orientations, or ages above 60 to better understand and address sexual side effects experienced by these populations.

It is also suggested that future researches assess similar possible effects on female sexual dysfunction as well in order to provide a better pathophysiological understanding as well as efficient treatment methods for females dealing with sexual side effects. Further studies on larger populations are required to investigate the possible effects of herbal medications such as *Alpinia galanga* extract on the sexual side effects of SSRIs.

The use of herbal medications such as *Alpinia galanga* needs to be assessed in patients with more severe levels of depression/anxiety as well to investigate whether the severity of the underlying mental illness can impact the effects of this medication. In this study, we found no significant relationship between the consumption of *Alpinia galanga* and AST, ALT, ALP, and creatinine laboratory values. We recommend future studies further investigate possible side effects of herbal medications such as *Alpinia galanga* to provide a better understanding of their possible benefits, risks, or usage in treatment. We also suggest future studies investigate and compare the possible efficacy and side effects of *Alpinia galanga* with other drugs such as sildenafil. It is hoped that by bringing more attention to subjects such as sexual function, enhancing the patient-physician relationship, and providing adequate education to people of different groups, discussions such as sexual function and activity will be easier and more fruitful. It is suggested that possible drug interactions and long-term and short-term side effects of herbal medications such as *Alpinia galanga* be studied as well before they are routinely prescribed to patients to enhance their sexual function.

## Conclusion

According to the results of this study as well as similar studies, the use of *Alpinia galanga* for the management and reduction of SSRI-associated sexual dysfunction has been promising. The reported effects, if further proven by future studies, may lead to a better understanding of the causes and mechanisms of medication-induced sexual dysfunction, as well as provide more effective methods of reducing or treating sexual side effects of different medications including psychotropic medications. It is hoped that more efficient treatment guidelines and protocols that effectively address possible side effects experienced by patients lead to better patient-physician communication, higher compliance, more fruitful treatment outcomes, and higher satisfaction rates among patients and clinicians alike which can, in turn, play a role in the reduction in the prevalence and burden of mental illnesses worldwide.

## Data availability statement

The original contributions presented in the study are included in the article/supplementary material, further inquiries can be directed to the corresponding author.

## Ethics statement

The studies involving human participants were reviewed and approved by the Research Ethics Committee of Mashhad University of Medical Sciences. The patients/participants provided their written informed consent to participate in this study. Written informed consent was obtained from the individual(s) for the publication of any potentially identifiable images or data included in this article.

## Author contributions

All authors contributed to the data collection, analysis, and manuscript preparation.

## Conflict of interest

The authors declare that the research was conducted in the absence of any commercial or financial relationships that could be construed as a potential conflict of interest.

## Publisher’s note

All claims expressed in this article are solely those of the authors and do not necessarily represent those of their affiliated organizations, or those of the publisher, the editors and the reviewers. Any product that may be evaluated in this article, or claim that may be made by its manufacturer, is not guaranteed or endorsed by the publisher.

## References

[1] Krasowska D Szymanek M Schwartz RA Myśliński W. Cutaneous effects of the most commonly used antidepressant medication, the selective serotonin reuptake inhibitors. J Am Acad Dermatol. (2007) 56:848–53. doi: 10.1016/j.jaad.2006.10.020, PMID: 17147971

[2] Ferguson JM. SSRI antidepressant medications: Adverse effects and tolerability. Prim Care Companion J Clin Psychiatry. (2001) 3:22–7. doi: 10.4088/pcc.v03n0105, PMID: 15014625 PMC181155

[3] Medford N Sierra M Baker D David A. Understanding and treating depersonalisation disorder. Adv Psychiatr Treat. (2005) 11:92–100. doi: 10.1192/apt.11.2.92

[4] AlBreiki M AlMaqbali M AlRisi K AlSinawi H Al Balushi M Al ZW. Prevalence of antidepressant-induced sexual dysfunction among psychiatric outpatients attending a tertiary care hospital. Neurosciences. (2020) 25:55–60. doi: 10.17712/nsj.2020.1.20190058, PMID: 31982896 PMC8015629

[5] Stahl SM. Mechanism of action of serotonin selective reuptake inhibitors. Serotonin receptors and pathways mediate therapeutic effects and side effects. J Affect Disord. (1998) 51:215–35. doi: 10.1016/s0165-0327(98)00221-3, PMID: 10333979

[6] Ebert D. Therapy with selective serotonin reuptake inhibitors (SSRI). Indications, uses and risks. Fortschr Med. (1996) 114:243–7. PMID: 8766793

[7] Cascade E Kalali AH Kennedy SH. Real-world data on SSRI antidepressant side effects. Psychiatry. (2009) 6:16–8. PMID: 19724743 PMC2719451

[8] Montgomery SA Baldwin DS Riley A. Antidepressant medications: A review of the evidence for drug-induced sexual dysfunction. J Affect Disord. (2002) 69:119–40. doi: 10.1016/S0165-0327(01)00313-5, PMID: 12103459

[9] Bala A Nguyen HMT Hellstrom WJG. Post-SSRI sexual dysfunction: A literature review. Sex Med Rev. (2018) 6:29–34. doi: 10.1016/j.sxmr.2017.07.002, PMID: 28778697

[10] Calabrò RS Cacciola A Bruschetta D Milardi D Quattrini F Sciarrone F. Neuroanatomy and function of human sexual behavior: A neglected or unknown issue? Brain Behav. (2019) 9:e01389. doi: 10.1002/brb3.1389, PMID: 31568703 PMC6908863

[11] Werneke U Northey S Bhugra D. Antidepressants and sexual dysfunction. Acta Psychiatr Scand. (2006) 114:384–97. doi: 10.1111/j.1600-0447.2006.00890.x 17087787

[12] Atmaca M. Selective serotonin reuptake inhibitor-induced sexual dysfunction: Current management perspectives. Neuropsychiatr Dis Treat. (2020) 16:1043–50. doi: 10.2147/NDT.S185757, PMID: 32368066 PMC7182464

[13] Rudkin L Taylor M Hawton K. Strategies for managing sexual dysfunction induced by antidepressant medication. Cochrane Database Syst Rev. (2004) 18:CD003382. doi: 10.1002/14651858.CD003382.pub2 15495050

[14] Lorenz T Meston C. Exercise improves sexual function in women taking antidepressants: Results from a randomized crossover trial. Depress Anxiety. (2013) 31:188–95. doi: 10.1002/da.22208, PMID: 24754044 PMC4039497

[15] Montejo AL Prieto N de Alarcón R Casado-Espada N de la Iglesia J Montejo L. Management strategies for antidepressant-related sexual dysfunction: A clinical approach. J Clin Med. (2019) 8:1640. doi: 10.3390/jcm8101640, PMID: 31591339 PMC6832699

[16] Williams K Reynolds MF. Sexual dysfunction in major depression. CNS Spectr. (2006) 11:19–23. doi: 10.1017/s1092852900026729 16871134

[17] Chouni A Paul S. A review on phytochemical and pharmacological potential of *Alpinia galanga*. Pharm J. (2017) 10:09–15. doi: 10.5530/pj.2018.1.2

[18] Ratnasooriya WD Jayakody JRAC. Effects of aqueous extract of Alpinia calcarata rhizomes on reproductive competence of male rats. Acta Biol Hung. (2006) 57:23–35. doi: 10.1556/ABiol.57.2006.1.3, PMID: 16646522

[19] Negm SH Ragheb EM. Effect of (*Alpinia officinarum*) hance on sex hormones and certain biochemical parameters of adult male experimental rats. J Food Dairy Sci. (2019) 10:315–22. doi: 10.21608/jfds.2019.55653

[20] Ranjbaran M Chizari M Matori PP. Prevalence of female sexual dysfunction in Iran: Systematic review and meta-analysis. J Sabzevar Univ Med Sci. (2016) 22:1117–25.

[21] Rhoden EL Telöken C Sogari PR Vargas Souto CA. The use of the simplified international index of erectile function (IIEF-5) as a diagnostic tool to study the prevalence of erectile dysfunction. Int J Impot Res. (2002) 14:245–50. doi: 10.1038/sj.ijir.3900859, PMID: 12152112

[22] Pakpour AH Zeidi IM Yekaninejad MS Burri A. Validation of a translated and culturally adapted Iranian version of the international index of erectile function. J Sex Marital Ther. (2014) 40:541–51. doi: 10.1080/0092623X.2013.788110, PMID: 24308814

[23] Ghassemzadeh H Mojtabai R Karamghadiri N Ebrahimkhani N. Psychometric properties of a Persian-language version of the Beck depression inventory--second edition: BDI-II-PERSIAN. Depression Anxiety. (2005) 21:185–92. doi: 10.1002/da.20070 16075452

[24] Beck AT Ward CH Mendelson M Mock J Erbaugh J. An inventory for measuring depression. Arch Gen Psychiatry. (1961) 4:561–71. doi: 10.1001/archpsyc.1961.01710120031004, PMID: 13688369

[25] Dadfar M Kalibatseva Z. Psychometric properties of the Persian version of the short beck depression inventory with Iranian psychiatric outpatients. Scientifica. (2016) 2016:8196463. doi: 10.1155/2016/8196463, PMID: 27293979 PMC4886104

[26] Rafiee M Seifi A. Reliability and validity of Beck anxiety scale in university students. J Thoughts Behav. (2013) 8:37–46.

[27] Beck AT Epstein N Brown G Steer RA. An inventory for measuring clinical anxiety: Psychometric properties. J Consult Clin Psychol. (1988) 56:893–7. doi: 10.1037/0022-006X.56.6.893 3204199

[28] Kaviani H Mousavi AS. Psychometric properties of the Persian version of Beck anxiety inventory (BAI). Tehran Univ Med J. (2008) 66:136–40.

[29] Hosten AO. BUN and creatinine In: Walker HK Hall WD Hurst JW, editors. Clinical methods: The history, physical, and laboratory examinations. 3rd ed. Boston: Butterworths (1990) 21250045

[30] Farahmand M Ramezani Tehrani F. The effect of medicinal plants in the treatment of sexual disorders: A narrative review. Iranian J Obstetr Gynecol Infertil. (2021) 24:87–102.

[31] Chen L Shi GR Huang DD Li Y Ma CC Shi M. Male sexual dysfunction: A review of literature on its pathological mechanisms, potential risk factors, and herbal drug intervention. Biomed Pharmacother. (2019) 112:108585. doi: 10.1016/j.biopha.2019.01.046, PMID: 30798136

[32] Banihani SA. Ginger and testosterone. Biomol Ther. (2018) 8:119. doi: 10.3390/biom8040119, PMID: 30360442 PMC6316093

[33] Qureshi S Shah AH Ageel AM. Toxicity studies on *Alpinia galanga* and Curcuma longa. Planta Med. (1992) 58:124–7. doi: 10.1055/s-2006-961412 1529022

[34] da Cruz AC Guerra NG de Souza KEBP de Castro Eleutério I da Silva LC Otoni EG. The action of herbal medicine on the libido: Aspects of nutritional intervention in increasing sexual desire. Forum Nutr. (2017) 42:29. doi: 10.1186/s41110-017-0051-0

[35] Luft MJ Dobson ET Levine A Croarkin PE Strawn JR. Pharmacologic interventions for antidepressant-induced sexual dysfunction: A systematic review and network meta-analysis of trials using the Arizona sexual experience scale. CNS Spectr. (2021). doi: 10.1017/S1092852921000377. [Epub ahead of print], PMID: 33843553

[36] Maleki-Saghooni N Mirzaeii K Hosseinzadeh H Sadeghi R Irani M. A systematic review and meta-analysis of clinical trials on saffron (*Crocus sativus*) effectiveness and safety on erectile dysfunction and semen parameters. Avicenna J Phytomed. (2018) 8:198–209. PMID: 29881706 PMC5987435

